# Can the impacts of cold-water pollution on fish be mitigated by thermal plasticity?

**DOI:** 10.1093/conphys/coaa005

**Published:** 2020-02-18

**Authors:** M A Parisi, R L Cramp, M A Gordos, C E Franklin

**Affiliations:** 1 School of Biological Sciences, The University of Queensland, Brisbane, Queensland 4072, Australia; 2 Department of Primary Industries (Fisheries), Wollongbar, New South Wales 2477, Australia

**Keywords:** Cold-water pollution, conservation, metabolic rate, phenotypic plasticity, swimming performance, temperature

## Abstract

Increasingly, cold-water pollution (CWP) is being recognised as a significant threat to aquatic communities downstream of large, bottom-release dams. Cold water releases typically occur during summer when storage dams release unseasonably cold and anoxic hypolimnetic waters, which can decrease the temperature of downstream waters by up to 16°C. Depending on the release duration, these hypothermic conditions can persist for many months. The capacity of ectothermic species to tolerate or rapidly adjust to acute temperature changes may determine the nature and magnitude of the impact of CWP on affected species. This study assessed the impacts of an acute reduction in water temperature on the physiological function and locomotor performance of juvenile silver perch (*Bidyanus bidyanus*) and examined their capacity to thermally compensate for the depressive effects of low temperatures via phenotypic plasticity. Locomotor performance (*U*crit and *U*sprint) and energetic costs (routine and maximum metabolic rate) were measured at multiple points over a 10-week period following an abrupt 10°C drop in water temperature. We also measured the thermal sensitivity of metabolic enzymes from muscle samples taken from fish following the exposure period. Cold exposure had significant depressive effects on physiological traits, resulting in decreases in performance between 10% and 55%. Although there was partial acclimation of *U*crit (~35% increase in performance) and complete compensation of metabolic rate, this occurred late in the exposure period, meaning silver perch were unable to rapidly compensate for the depressive effects of thermal pollution. The results of this study have substantial implications for the management of cold water releases from large-scale dams and the conservation of native freshwater fish species, as this form of thermal pollution can act as a barrier to fish movement, cause reduced recruitment, ecological community shifts and disruptions to timing and success of reproduction.

## Teaser

Many Australian fish species are subject to thermal pollution from unseasonably cold water released from large storage dams. This study assessed the impacts and rate of physiological compensation to low temperatures following a simulated cold-water pollution event on juveniles of critically endangered silver perch (*Bidyanus bidyanus*).

## Introduction

The role of temperature as a key environmental driver of physiological performance in ectotherms has been recognised for decades; however, the capacity for species to adjust and respond to temperature changes within their environment is increasingly acknowledged as an important trait that can be used to model the threats posed to organisms from anthropogenic climate change (Seebacher *et al*., 2015). But it is not just climate warming that threatens many freshwater ectotherms. Globally, the thermal regime of rivers, inlets and estuaries has been substantially altered by the construction of large-scale dams (Olden and Naiman, 2010). Water impounded behind large dams thermally stratifies into warm surface (epilimnion) layers and cold bottom (hypolimnion) layers (Neel, 1963). Water release patterns from large dams can markedly influence the downstream thermal environment—with changes including increased winter temperatures, depressed summer temperatures, reduced intra- and inter-annual variability, reduced diel variability and delayed seasonal peaks (reviewed by Lugg and Copeland, 2014). Of these, the release of cold water from hypolimnetic layers of large dams, termed cold-water pollution (CWP), has been identified as a key threatening process in freshwater systems (Lugg and Copeland, 2014). The effects of CWP can extend for up to 350 km downstream from the release point of the dam causing reductions in water temperature of up to 16°C (Preece, 2004; Lugg and Copeland, 2014) and are likely to have contributed substantially to the decline of a large number of native fish species (Boys *et al*., 2009).

While the effects of CWP on downstream water temperatures have been acknowledged for almost half a century (e.g. Lake, 1967), only relatively recently have the biological impacts of these events been brought to light by studies demonstrating long-term negative effects on Australian fish populations (e.g. Preece and Jones, 2002; Astles *et al*., 2003; Lyon *et al*., 2008). Several fish species native to the highly regulated Murray–Darling Basin (MDB) in Australia have been reported to show reduced growth rates and increased mortality at water temperatures consistent with CWP events (Ryan *et al*., 2002; Astles *et al*., 2003; Ryan and Preece, 2003). Todd *et al*. (2005) suggested that CWP is responsible for the local extinction of several populations of Murray cod (*Maccullochella peelii peelii*) as a consequence of its effects on post-spawning larval mortality. Although fish may experience similarly low water temperatures during winter, it is possible that the rate and magnitude of these unseasonably cold releases limit the capacity of fish to physiologically compensate for the depressive effect of low temperatures on performance. While acute exposure to low temperatures can negatively affect many tropical and temperate fish species (Ryan *et al*., 2002; Astles *et al*., 2003; Lyon *et al*., 2008; Rodgers *et al*., 2014), many ectotherms have the capacity to thermally compensate for chronic shifts in water temperature (Seebacher *et al.,* 2015).

Most environments experience some degree of thermal heterogeneity, whether it be on diel, seasonal or geological time scales. While genetic adaptation can match organism phenotypes to stable environmental conditions experienced over the course of many generations, environmental variability experienced within a generation can influence organism phenotype both irreversibly (developmental plasticity) and reversibly [reversible plasticity (acclimation)] (Beaman *et al*., 2016). For many ectotherms, performance is optimised over a range of environmental temperatures that reflect the temperature of the immediate environment; exposure to temperatures outside of the optimal thermal range results in a reduction in performance (Huey and Kingsolver, 1989). Predictable or chronic environmental changes, like those occurring with temperature over seasonal time scales, can induce a reversible physiological re-adjustment of performance optima that serve to better match the organism’s phenotype to the prevailing conditions (reversible acclimation) (Schulte *et al*., 2011). However, thermal acclimation may be maladaptive in highly thermally heterogeneous environments where the costs of acclimating may mean that by the time the response is fully developed, the environment may have changed resulting in an environment–phenotype mismatch (Beaman *et al*., 2016). In such cases, organisms may be better served by a broader thermal performance optima or thermal insensitivity (independence) over the range of temperatures experienced in that environment (Wilson and Franklin, 2002; Niehaus *et al.,* 2012; Kern *et al.,* 2015). While thermal phenotypic plasticity allows ectotherms to occupy a diverse array of thermally heterogeneous environments, the rate of temperature change, the degree of thermal variability within the environment, the thermal history of the animal and interactions with other important environmental cues may determine both the capacity for, and extent of, physiological compensation possible (Wilson and Franklin, 2002; Guderley, 2004; Niehaus *et al.,* 2006). Consequently, although fish may experience low water temperatures during winter, it is possible that the rate and magnitude of unseasonably cold water releases from thermally stratified dams limit the capacity of fish to physiologically compensate for the depressive effect of low temperatures on performance.

Many physiological traits show a capacity for thermal plasticity in fish (e.g. Sisson and Sidell, 1987; Guderley and Blier, 1988; Hammill *et al*., 2004; Little and Seebacher, 2013; Cai *et al*., 2014; Fangue *et al*., 2014; Sandblom *et al*., 2014), with locomotor and metabolic parameters being routinely examined not only because of their ease and repeatability of measurement, but also because they are strong proxies for fitness in animals (Arnold, 1983; Irschick and Garland, 2001). The regulation of metabolism is important for thermal acclimation because it determines the individual’s capacity to maintain stable energy production in the face of thermal variability and, therefore, the capacity to maintain consistent performance across other physiological traits like locomotion (Scott and Johnston, 2012; Beaman *et al*., 2016). Consequently, much of the cellular machinery underlying both locomotor performance and metabolic rate is also thermally plastic (Guderley, 2004) so compensatory responses to temperature on a biochemical level may reflect or constrain the thermal plasticity of whole-animal level traits (Seebacher *et al*., 2003).

In highly regulated freshwater environments like the MDB, the ability of fish to maintain performance over a range of thermal conditions may determine their capacity to traverse large-scale fish passage structures (fishways or fish passes) and undertake large-scale migrations. Anthropogenic barriers (dams, weirs, culverts) to fish migration are responsible for the decline of hundreds of freshwater fish species globally (Kingsford, 2000; Baumgartner *et al*., 2014; Lugg and Copeland, 2014; Watson *et al.,* 2018), and as a result billions of dollars have been invested in providing fish passage structures that enable fish to migrate around manmade instream barriers (Bunt *et al*., 2012; Goodrich *et al.,* 2018). However, the usefulness of these structures for fish passage may be reduced if CWP-associated conditions preclude fish species unable to thermally adjust performance to compensate for the low environmental temperatures. Moreover, the timeframe over which thermal compensation for acute reductions in water temperature occurs may limit the usefulness of thermal compensation for facilitating fish passage in thermally polluted environments. Few studies have explored the rate of physiological compensation following acute exposure to low temperatures. The ability of fish to respond rapidly to acute reductions in temperature through acclimation would clearly be advantageous during CWP events and may determine those species or life history stages most tolerant of CWP-associated stresses. Conversely, mounting an acclimation response following an acute reduction in water temperature may be disadvantageous if environment–phenotype mismatches occur once temperatures return to ‘normal’ or animals successfully move out of thermally polluted areas.

The aim of this study was to investigate how physiological performance traits of fish respond following an acute reduction in water temperature equivalent to that experienced during CWP events in river systems and over a similar time scale. We measured the swimming performance (sprint [*U*sprint] and critical [*U*crit]) and energy expenditure (routine and maximal metabolic rates) of juvenile silver perch (*B. bidyanus*) at intervals over the experimental period and quantified the time course of physiological adjustments to these traits. We also examined key metabolic enzyme activities at the completion of the exposure period to assess the physiological impacts of acute/chronic cold-temperature exposure at the biochemical level. Silver perch are an Australian fish species native to the MDB where they experience seasonal water temperatures that range from 8°C to 30°C (WaterNSW, 2016). Silver perch populations in the MDB have suffered significant declines (Rowland, 2004), and consequently the species is now listed as ‘Critically Endangered’ under the Australian Environment Protection and Biodiversity Conservation Act 1999. Given that the natural environmental temperatures experienced by this species vary substantially but largely predictably over the course of a year (WaterNSW, 2016), we hypothesised that *B. bidyanus* would be able to compensate physiological performance following an acute reduction in water temperature similar in scale to that experienced during a CWP water release.

## Materials and methods

Juvenile silver perch (n = 100; total length, 5–15 cm; mass, 3–25 g) were purchased in September 2015 from a commercial hatchery (Ausyfish, Queensland, Australia) and housed in two 1000-l recirculating aquarium systems, each with 12 separate 60-l tanks. Fish were stocked at a density of 5–7 individuals per tank and water temperatures were maintained at their hatchery rearing temperature of 24°C using aquarium heater/chiller units (TK1000, Teco, Ravenna Italy). The photoperiod was set to a 12-h light and 12-h dark cycle. Tanks were supplied with carbon-filtered Brisbane tap water, and water changes were conducted continuously via a drip feed system. Silver perch were fed commercial fish food pellets (Hikari Tropical Micro Wafers, Kyorin Food Industries, Japan) daily. Prior to experimentation, individual fish were tagged with visible implant elastomer tags (Northwest Marine Technology, Washington, USA) and given 7 days to recuperate before experimentation commenced. Fish were re-tested throughout the experiment, however, never for the same physiological performance metric 2 weeks in a row.

### Experimental design

Following the tagging and recovery period, the water temperature in one 1000-l system (12 tanks, *n* = 50 fish) was lowered over a 6-h period to 14°C (‘cold’ treatment). An additional chiller (HC1000A; Hailea, GuangDong, China) was added to this system to maintain consistent temperature. The ‘warm’ treatment tanks were maintained at 24°C. The selection of treatment temperatures were based on a field study conducted by Astles *et al*. (2003), which identified these as temperatures representative of natural ambient river temperature and post-release water temperature in the Macquarie River downstream of Burrendong Dam (32°40’S 149°09’E) in the MDB during summer. Ryan and Preece (2003) highlighted the rapid rate at which temperature can decrease during a CWP event in the MDB. Physiological performance metrics were measured in a subset of fish (*n* = 12 per trait) from each treatment at weeks 0, 1, 2, 3, 5, 7 and 9 at their acclimation temperatures. At the end of the acclimation period (week 10), traits were measured at both the acclimation temperature and the contrasting temperature. Different individuals were used for each metric, except for maximum oxygen consumption rates [maximal metabolic rate (MMR)], which were measured immediately after critical swimming speed (*U*crit). All fish were fasted for 24 h prior to measurement.

### Swimming performance

Swimming performance measurements were conducted in a thermostatically controlled 185-l recirculating swimming flume (Loligo, Tjele, Denmark; swimming chamber dimensions = L × W × H, 87.5 × 25 × 25 cm). The flume was calibrated using a Prandtl-pitot tube to take point velocity measurements in the middle of the length of the swimming chamber (Kern *et al*., 2018). This was done at several speed settings and used to determine the average cross-sectional velocity in the swim chamber. A calibration curve was then used to set the water velocity within the swim chamber. Water temperatures were maintained using an aquarium chiller (Hailea HC-1000A, Guangdong, China).

All *U*crit trials were conducted using groups of four similarly sized fish. *U*crit was measured following Brett (1964) and Rodgers *et al*. (2014). Briefly, fish were placed into the flume and water velocity was increased to a starting velocity of 10 cm s^−1^ for 5 min. Water velocities were then increased by 3 cm s^−1^ every 5 min until the fish fatigued (defined as the fish resting against the back wall of the swim chamber for 3 s). Sprint swimming speed (*U*sprint) measurements were made following the protocol described by Starrs *et al*. (2011). Unlike *U*crit, *U*sprint trials were conducted with individually swum fish. As with *U*crit, fish were placed into the swimming flume and the water velocity was increased to a starting velocity of 10 cm s^−1^ for 5 min. Water velocity was then increased by 3 cm s^−1^ every 10 s until the fish fatigued. *U*crit and *U*sprint were calculated using the equation from Brett (1964):

$$\begin{equation*}U\textrm{crit}\ \textrm{or}\ U\textrm{sprint} = U_f + [U_i (T_f / T_i)]\end{equation*}$$

where *U_f_* is the highest sustained water velocity achieved by the fish (cm s^−1^), *U_i_* is the increment that water velocity was increased by (3 cm s^−1^), *T_f_* is the time swam during the final increment and *T_i_* is the entire velocity interval (*U*crit: 300 s or *U*sprint: 10 s).

### Metabolic rate

Routine metabolic rate (RMR) and MMR were measured using closed system respirometry. Respirometers (Sistema KLIP IT containers, Auckland, New Zealand) ranging from 1000 to 4000 ml were connected to a water pump (Eheim, Deizisau, Germany) to create a closed-circuit recirculating loop to ensure mixing throughout the respirometer (Clark *et al*., 2013). Respirometers were placed into a water bath at the appropriate test temperature (either 14°C or 24°C). Oxygen concentration (as % air saturation) was measured non-invasively using a Fibox 3 and oxygen sensitive sensor spots (PreSens, Regensburg, Germany) affixed to the inner wall of the respirometer. For measures of RMR, fish were placed into the respirometers and given 30 min to settle before measurements began. Oxygen concentrations inside the respirometer were recorded every 10 min for 60 min. MMR was measured immediately following the measurement of *U*crit. Once fish had fatigued, they were immediately transferred into respirometers and aquatic oxygen concentrations were measured every min for 15 min. RMR and MMR were calculated using the equation

$$\begin{equation*} MR = -1 \times \Delta O_2 \times V \times \beta O_2\end{equation*}$$

where ΔO_2_ is the rate of change of oxygen saturation within the respirometer (as % air saturation per hour), V is the volume of the respirometer less the mass of the fish (assuming a density of 1 g ml^-1^) and βO_2_ is the solubility of oxygen in water at each treatment temperature (7.28 at 14°C and 5.91 at 24°C; Cameron, 1986).

### Q_10_ calculations

The temperature coefficient (Q_10_) over the thermal range 14–24°C was calculated for swimming performance and metabolic rates using the equation

$$\begin{equation*} Q_{10} = (R_2 / R_1)^{10/(T_2-T_1)}\end{equation*}$$

where R_2_ and R_1_ are the means for each performance trait at respective temperatures (T_2_ and T_1_).

### Metabolic enzyme assays

At the end of the 10-week acclimation period, a subset of fish (*n* = 10 per treatment) were euthanased in freshwater containing Tricaine methane-sulfonate buffered with NaH_2_CO_3_ (Tricaine-S 250 mg l^−1^; Western Chemical, Washington, USA). A sample of white muscle was dissected, snap frozen on dry ice and stored at −80°C. Muscle tissue (0.05–0.1 g) was homogenised in nine volumes of extraction buffer (pH 7.5; 50 mmol l ^−1^ imidazole, 2 mmol l^−1^ MgSO4, 5 mmol l^−1^ EDTA, 1 mmol l^−1^ glutathione, 0.1% Triton). The activity of lactate dehydrogenase (LDH), citrate synthase (CS) and cytochrome *c* oxidase (CCO) was measured in a spectrophotometer (Beckman Coulter DU 800, California, USA) with a Peltier controlled cuvette holder following Seebacher *et al*. (2003). Assays were conducted at both 14°C and 24°C for all fish and all three enzymes.

## Statistical analysis

All analyses were performed using the statistical package R (R Core Team, 2013) within the Rstudio platform (version 0.98.1103). Linear mixed effects models were used to examine the effects of test and acclimation temperatures and acclimation time on each performance trait and growth rates using the lmerTest package (Kuznetsova *et al*., 2017; Bates *et al*., 2015). ‘Acclimation treatment’ (14°C or 24°C) and ‘test temperature’ were treated as fixed factors and ‘week’ was treated as an ordered factor for analysis of the time course of exposure. ‘Fish ID’ nested within ‘Tank’ was included as a random effect to account for potential errors associated with sampling different individuals from the same tank. ‘Total length’ and ‘body mass’ were included as covariates for swimming performance and metabolic rate analyses, respectively. Metabolic rate data were log transformed to meet underlying model assumptions. For metabolic enzyme analyses, ‘Fish ID’ was nested within ‘Tank’ to account for repeated measurements on the same individual. Model selection was performed using the ‘step’ function in the lmerTest package. Least squared means comparisons were used to determine differences between acclimation/test temperatures where a significant F test indicated an interaction between the two factors. All data are presented as means ± s.e.

## Results

### Growth

Acclimation temperature had a significant effect on growth rates over the 10-week exposure period. At the end of the experimental period, animals in the 24°C acclimation treatment were longer (*t* = 18.1, *P* < 0.001) and heavier (*t* = 14.3, *P* < 0.001) than those in the 14°C acclimation treatment (Table 1).

**Table 1:** Total length and mass of juvenile silver perch, *B. bidyanus*, for 14°C and 24°C temperature treatments before and after the 10-week exposure period (*n* = 36 per treatment)

	**Initial**		**Final**	
**Treatment temperature (°C)**	**Total length (cm)**	**Mass (g)**	**Total length (cm)**	**Mass (g)**
14	9.3 ± 0.3	9.6 ± 0.9	9.4 ± 0.3	9.9 ± 1.1
24	9.5 ± 0.3	10.0 ± 1.0	11.2 ± 0.3	15.6 ± 1.4

Data shown as mean ± s.e.

**Figure 1:**
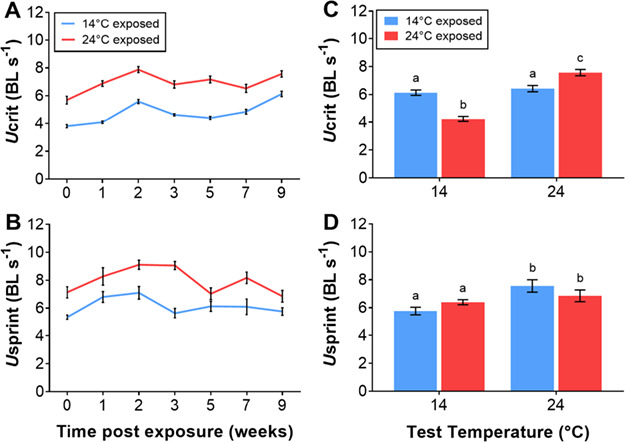
Critical (*U*crit) (**A**) and sprint (*U*sprint) (**B**) swimming speed (body lengths s^−1^) of juvenile silver perch, *B. bidyanus*, in 14°C or 24°C temperature treatments over exposure time (weeks). Effect of test temperature on *U*crit (**C**) and *U*sprint (**D**) of juvenile silver perch, acclimated to 14°C or 24°C for 10 weeks and tested acutely at both 14°C and 24°C. Data are presented as mean ± s.e., and there were 12 fish tested per treatment. Treatments with the same lowercase letter are not significantly different.

### Swimming performance

Prolonged exposure to 14°C resulted in an overall poorer performance for both *U*crit (*F*_1_, _11.6_ = 327.9, *P* < 0.001; Fig. 1A) and *U*sprint (*F*_1, 12.4_ = 40.9, *P* < 0.001; Fig. 1B) at almost all time points relative to those maintained and tested at 24°C. There was a significant interaction between acclimation temperature and time for both *U*crit and *U*sprint (*F*_6, 99.1_ = 3.7, *P* = 0.002; *F*_6, 101.2_ = 2.4, *P* < 0.001; Fig. 1A and B). In fish tested at the end of the 10-week exposure to 14°C (compared with fish maintained at 24°C), there was no significant effect of acclimation temperature (*F*_1,8.13_ = 0.47, *P* = 0.51) on *U*crit, but there was a significant main effect of test temperature (*F*_1,10.69_ = 297.89, *P* < 0.001) and an interaction between acclimation temperature and test temperature (*F*_1,13.14_ = 54.11, *P* < 0.001; Fig. 1C). Acute exposure to 14°C had significant depressive effects on *U*crit and *U*sprint for fish maintained at 24°C (Fig. 1C and D). Fish acclimated to 14°C for 10 weeks and tested at 14°C had a significantly higher *U*crit than fish maintained at 24°C and acutely exposed to 14°C, suggesting some thermal compensation had occurred over the course of the exposure period. However, the *U*crit of fish maintained and tested at 24°C for 10 weeks remained significantly higher than that of fish maintained and tested at 14°C, suggesting that thermal compensation was only partial. The Q_10_ values for *U*crit (14°C–24°C) for the cold and warm acclimation groups were 1.05 and 1.78, respectively. For *U*sprint, performance was significantly lower when tested at 14°C than at 24°C (*F*_1,13.72_ = 7.12, *P* = 0.019) irrespective of acclimation temperature (*F*_1,9.08_ = 1.48, *P* = 0.254; Fig. 1D). The Q_10_ (14°C–24°C) values for *U*sprint at the end of the acclimation period for the cold and warm treatment groups were 1.31 and 1.07, respectively.

### Metabolic rate

**Figure 2:**
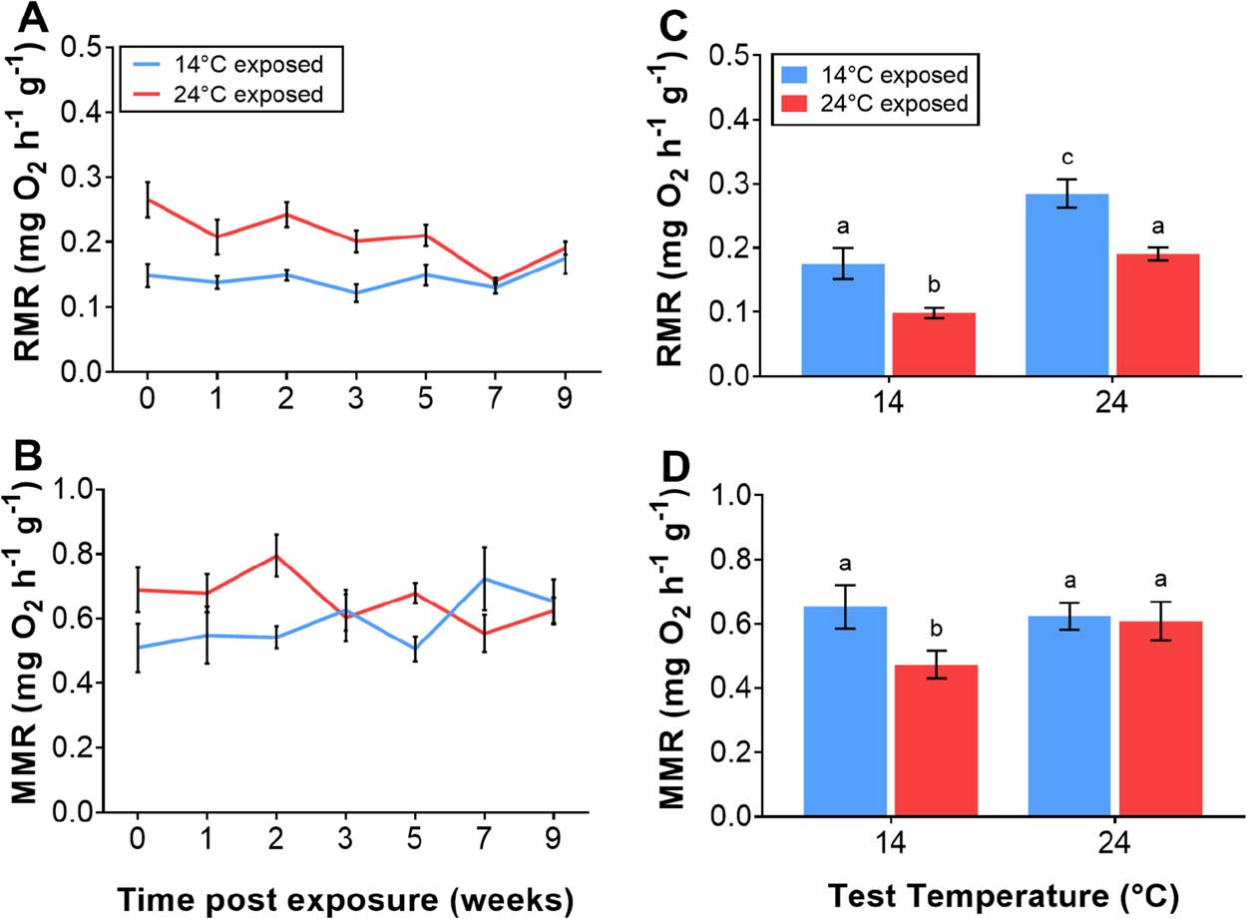
Rate of change in (**A**) routine (RMR) and (**B**) maximal (MMR) oxygen consumption (mg O_2_ h^−1^ g^−1^) of juvenile silver perch, *B. bidyanus*, acutely exposed then maintained at 14°C or 24°C over the exposure period (weeks). The effect of test temperature on RMR (**C**) and MMR (**D**) of juvenile silver perch, exposed to either 14°C or 24°C for 10 weeks and tested acutely at both 14°C and 24°C. Data are presented as mean ± s.e., and there were 12 fish measured per treatment. Treatments with the same lowercase letter are not significantly different.

In order to examine the rate of change in metabolic rates following exposure to 14°C, RMR and MMR were measured multiple times over the exposure period. There was a significant main effect of acclimation temperature and time post exposure (*F*_1, 59.74_ = 42.75, *P* < 0.001 and *F*_1,96.77_ = 6.49, *P* = 0.012, respectively) on RMR (Fig. 2A). In addition there was a strong interaction between acclimation temperature and time for RMR due to a decrease in the RMR of fish in the 24°C acclimation treatment over time (*F*_1, 101.01_ = 8.27, *P* = 0.005; Fig. 2A). RMR of 14°C exposed fish did not vary significantly over the acclimation period. MMR varied significantly between acclimation treatments (*F*_1, 9.4_ = 13.4, *P* = 0.004), but there was no main effect of exposure time (*F*_6, 84.2_ = 1.9, *P* = 0.09; Fig. 2B). There was, however, a significant interaction between acclimation group and exposure time (*F*_6, 78.9_ = 2.7, *P* = 0.04), with MMR decreasing slightly over time in the 24°C acclimation group and increasing slightly in the 14°C acclimation group.

Following the 10-week exposure, there was a significant main effect of both test temperature (*F*_1,14.3_ = 88.61, P < 0.001) and acclimation temperature (*F*_1,10.82_ = 27.07, P < 0.001) on RMR, with fish in the 14°C acclimation treatment having a higher RMR at both test temperatures than fish from the 24°C acclimation treatment (Fig. 2C). There was no significant interaction between acclimation temperature and test temperature. The Q_10_ values for RMR for the cold and warm treatment groups were 1.62 and 1.93, respectively. There was no significant effect of acclimation temperature on final MMR (*F*_1, 5.2_ = 3.5, *P* = 0.12) but there was a significant effect of test temperature, with MMR lower for fish tested at 14°C from the 24°C acclimation treatment (*F*_1, 7.6_ = 16.57, *P* = 0.004; Fig. 2D). The Q_10_ values for MMR at the end of the acclimation period for the cold and warm acclimation groups were 0.93 and 1.3, respectively.

### Enzyme assays

**Figure 3:**
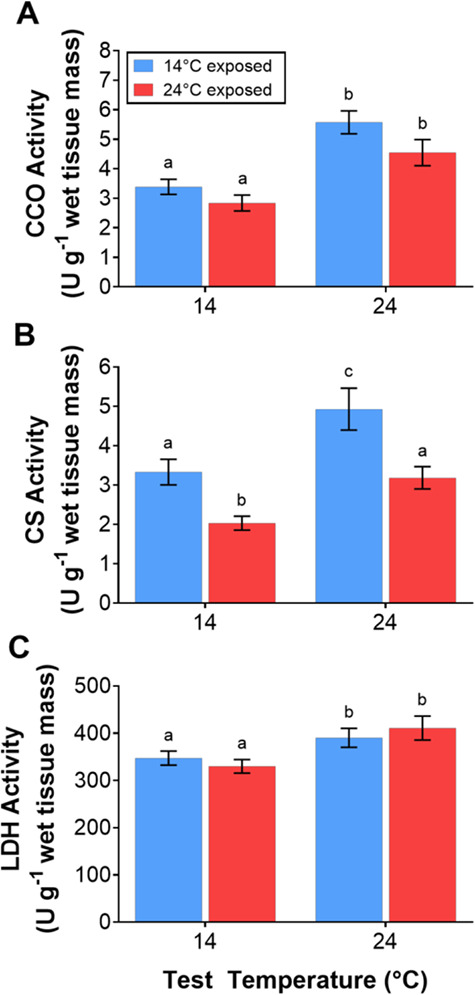
*In vitro* activity of (**A**) CCO, (**B**) CS and (**C**) LDH from white muscle tissue of juvenile silver perch, *B. bidyanus*, exposed to 14°C or 24°C for 10 weeks and tested at both 14°C and 24°C (*n* = 10 per treatment per test temperature). Data are presented as mean ± s.e. Treatments with the same lowercase letter are not significantly different.

There was no effect of acclimation temperature on muscle CCO and LDH activities (CCO: F_1, 21.3_ = 0.02, P = 0.88; LDH: F_1, 25_ = 1.7, P = 0.2), but there was a significant effect of test temperature (CCO: F_1, 18_ = 99.2, P < 0.001; LDH: F_1, 18_ = 26.3, P < 0.001; Fig. 3A and C). Both CCO and LDH activities were reduced at 14°C relative to their activities at 24°C. CS activity was significantly affected by both test temperature (F_1, 29.28_ = 17.69, P < 0.001) and acclimation temperature (F_1, 5.35_ = 12.6, P = 0.015) with higher activity in tissues from the 14°C acclimated fish at both test temperatures (Fig. 3B). CS activity was greatest when tested at 24°C.

## Discussion

Both acute and prolonged exposure to cold temperatures, similar in magnitude to those occurring during CWP events, had substantial negative effects on physiological performance in juvenile silver perch (*B. bidyanus*). Acute exposure to 14°C water depressed all performance traits measured; however, the capacity for thermal compensation over the subsequent 10-week exposure period differed across the physiological and biochemical traits measured. Sprint swimming speed (*U*sprint), muscle LDH and CCO activities showed no evidence of thermal compensation, while critical swimming speed (*U*crit), RMR, MMR and muscle CS activities showed at least some degree of thermal compensation. In addition, prolonged exposure to low temperatures substantially reduced growth rates. Although juvenile silver perch had some capacity to compensate for the persistent effect of low temperature on *U*crit, RMR and MMR, this capacity did not manifest until at least 7 weeks of exposure, suggesting that CWP-like reductions in water temperature prevent fish from rapidly compensating for the depressive effects of low temperature on performance. These findings have substantial implications for the management of critically endangered silver perch downstream of large dams that release water from hypolimnetic regions.

Silver perch demonstrated some capacity to thermally compensate *U*crit performance following prolonged exposure to 14°C water, increasing their *U*crit performance by ~35%. However, even after 10 weeks, fish held at 14°C were still performing worse (~20% lower) than fish maintained and tested at 24°C. Although *U*crit was still significantly lower at 14°C than at 24°C after 10 weeks of exposure, these results show that in silver perch, swimming performance can undergo a reasonable degree of thermal acclimation over the experimental time frame. However, the capacity to compensate was not evident until 7 weeks post-exposure. The results suggest that given enough time, thermal plasticity may completely compensate for the negative effects of low temperatures on performance; however, the rate at which this occurred in silver perch was not sufficient to allow fish to avoid impacts on associated physiological processes like growth rates. Although not directly examined in this study, sustained impacts on swimming performance with CWP are likely to impair fish foraging efficiency and predator avoidance capacities (Donaldson *et al.,* 2008). A delay in the development of compensatory responses following an acute decrease in water temperature may be due to the rapid nature of the temperature change, the large magnitude of change and the extent of biochemical remodelling required to buffer the effects of low temperatures.

The acute effects of cold water exposure on fish swimming performance are partly the result of impaired energy supply to the muscles (McKenzie, 2011). Since metabolic rate is reflective of energy usage over time, a lowered RMR following exposure to cold temperatures likely reflects the direct thermodynamic actions of low temperatures on energetic processes (McKenzie, 2011). However, a capacity to increase metabolic rate with prolonged exposure to low temperatures (‘metabolic cold acclimation’) has been seen in many fish species (Guderley, 2004) and was apparent for both RMR and MMR in silver perch in this study. The abbreviated measurement protocol we used for metabolic rate may have masked some responses recorded and potentially underestimated the magnitude of the effect of temperature on fish in our study. However, we believe the measures are still relative as all procedures remained consistent among treatments.

Thermal compensation of energy-producing processes occurs through changes in mitochondrial volume density (Guderley, 1990), enhanced oxidative capacities of mitochondria in muscles (Guderley, 1996), structural changes to cell membranes and changes in muscle enzyme catalytic capacities (Hazel, 1984). Consistent with this, CS activity in silver perch white muscle showed evidence of thermal compensation with improved activity at 14°C in the tissues of 14°C acclimated fish. Increased CS activity has been hypothesised to serve as a winter compensatory mechanism during seasonal acclimation in alligators (Seebacher *et al*., 2003) and in a number of other fish species, such as green sunfish (*Lepomis cyanellus*), smallmouth bass (*Micropterus dolomieui*) (Kolok, 1991) and Atlantic cod (*Gadus morhua*) (Martinez *et al*., 1999). Although increased activity of both CCO and LDH often accompanies winter acclimation in other fish species (e.g. Martinez *et al*., 1999; Seebacher *et al*., 2003), there was no evidence of acclimation of CCO and LDH in silver perch after the 10-week acclimation period. This suggests that either these processes are not thermally plastic in silver perch or the rapid nature of the temperature change may have precluded the development of compensatory adjustments in these enzymes.

The results of this study suggest that there is likely to be a significant ecological impact of CWP on silver perch given the magnitude of the physiological impacts observed, which raises a number of important management considerations. Firstly, cold water exposure significantly impaired fish swimming performance meaning that CWP is likely to act as a thermal barrier to fish movement. In addition, silver perch have been shown to avoid cold water (Astles *et al.,* 2003) suggesting they may avoid moving into CWP-affected river reaches. CWP may impact the capacity for silver perch not only to undertake crucial meso-scale activities such as foraging, finding mates and evading predators, but also to undertake seasonal large-scale migrations particularly in heavily regulated waterways. Fish passage structures that are designed to promote migratory fish passage over dams and weirs pose considerable physiological challenges, particularly for juvenile fish. If water temperature reductions coincide with migratory movements, then the performance challenges that these passage structures impose may be compounded by the thermodynamic effects of low temperature on fish performance. The regulation of fish passage structures (water discharge capacities) in areas affected by CWP needs to consider the physiological impacts of low water temperatures on the swimming capacities of target species.

Secondly, this study highlights the importance of the rate of acclimation in determining the long-term impacts of thermal pollution on fish. Although silver perch demonstrated a capacity to thermally compensate some physiological traits, it was not rapid and was dependent on the period of exposure and particular trait measured. The capacity for thermal plasticity in fitness-critical traits and their rate of acclimation are potentially significant factors underpinning the broader-scale responses of fish communities in environments where they may be subject to random, rapid and/or extreme changes in temperature. In some circumstances, thermal phenotypic plasticity may offset the depressive effects of low temperatures and promote the maintenance of performance across a range of temperatures. However, the time-course for acclimation of a particular trait will determine whether or not it is ultimately beneficial (Rogers *et al*., 2004). If the environmental condition has changed by the time the response is fully developed, an environment-phenotype mismatch may result in a disadvantage to the individual (Beaman *et al*., 2016). In addition, the energetic cost of acclimation needs to be considered, as different rates of acclimation have a greater energetic cost (Sandblom *et al*., 2014). For example, there is a greater overall energetic cost of acclimation for slow-acclimating species, affecting fitness-related traits and potentially influencing competition between coexisting species that are ‘slow’ and ‘fast’ acclimating (Sandblom *et al.*, 2014). Similarly, Donelson *et al*. (2011) found that growth and body condition came at a cost for fish acclimated to elevated temperatures. It is clear that the ability to acclimate, the time course for acclimation and the associated costs are all critical factors to be considered when assessing the potential impacts of CWP, with the latter two relatively unstudied and a fertile avenue for future studies.

Species-specific acclimation abilities and different costs of acclimation can have ecological implications for fish communities in CWP areas. Many invasive fish species are regarded as having a broader thermal breadth, which may provide them with a competitive advantage over native species. For example, invasive common carp, which now account for up to 90% of fish biomass in some areas of the MDB (Commonwealth Environmental Water Office, 2016), have enhanced swimming performance at low temperatures (Fleming *et al*., 1990) and mosquito fish have the ability to acclimate swimming performance over a 12°C temperature range (Hammill *et al*., 2004). CWP events coupled with the presence of competitive invasive species could create additional pressures for native species like silver perch, with consequences including re-distribution of species, reduced recruitment to adult populations, disruptions to timing and success of reproduction and declines in populations (Astles *et al*., 2003; Boys *et al*., 2009; Lugg and Copeland, 2014).

It is evident that there is a need to manage CWP events in freshwater ecosystems to minimise the current impacts on freshwater fish, but how this will be achieved needs further investigation. Dams fitted with multi-level offtakes may be able to release water from higher in the impoundment where water temperatures more closely reflect downstream temperatures. However, the utility of this approach to manage CWP is dependent on the presence of appropriate infrastructure, which is economically costly, and the absence of algae (Lugg and Copeland, 2014). Furthermore, thermal breadth and acclimation ability likely differs greatly between both native and exotic fish species (Fleming *et al*., 1990; Hammill *et al*., 2004; Lyon *et al*., 2008). Silver perch may be particularly vulnerable to CWP, or comparatively insensitive, therefore investigating thermal limits for a range of species is a crucial step in understanding the ecosystem-wide impacts of CWP and inclusively managing remediation efforts. Field studies may also provide an insight into other mechanisms fish are employing to compensate during these events (e.g. behavioural modifications). Finally, further investigations into the combined effects of other stressors (e.g. low dissolved oxygen levels) with low temperature may provide a more holistic view of how CWP events influence downstream fish populations.
