# Correction to: Can the impacts of cold water pollution on fish be mitigated by thermal plasticity?

**DOI:** 10.1093/conphys/coac012

**Published:** 2022-03-11

*Conserv Physiol* 8(1): coaa005; doi: 10.1093/conphys/coaa005

In the originally published version of this manuscript, there were errors in metabolic rate units. On p.6, in Fig. 2, these should read: “ml” throughout instead of “mg” throughout. And in the legend to Fig. 2 this should read: “Rate of change in (**A**) routine (RMR) and (**B**) maximal (MMR) oxygen consumption (ml O_2_ h^−1^ g^−1^) of juvenile silver perch,” instead of: “Rate of change in (**A**) routine (RMR) and (**B**) maximal (MMR) oxygen consumption (mg O_2_ h^−1^ g^−1^) of juvenile silver perch,”. These errors are now corrected online.

